# Multivariate statistical analysis of chemical components, origin, variety, and sensory quality of domestic cigar tobacco leaves

**DOI:** 10.3389/fchem.2026.1910376

**Published:** 2026-09-23

**Authors:** Cai Wen, An Hongyue, Liu Jie, Zhang Qianying, Huang Mingyue, Chai Zhishun, Zhu Beibei, Huo Danqun, Li Dongliang

**Affiliations:** 1 China Tobacco Sichuan Industrial Co., Ltd., Chengdu, China; 2 School of Bioengineering, Chongqing University, Chognqing, China

**Keywords:** chemical component, cigar tobacco leaves, mass spectrometry, principal component analysis, sensory quality

## Abstract

**Introduction:**

To achieve precise quality control and targeted optimization of cigar tobacco leaves, this study established an integrated analytical framework combining multi-source chemical detection with multivariate statistical analysis, using domestic cigar tobacco leaves from 9 origins and 3 varieties (27 samples in total).

**Methods:**

Gas chromatography-mass spectrometry (GC-MS/MS) was employed for untargeted semi-quantitative profiling of volatile components. Liquid chromatography-tandem mass spectrometry (LC-MS/MS/MS) was used for the quantitative determination of characteristic alkaloids and nitrogen-containing heterocycles. Biochemical assay kits were used to measure total protein and total starch contents. Based on the obtained multi-dimensional chemical data, principal component analysis (PCA) was applied for dimensionality reduction and visualization to explore the distribution patterns of tobacco leaves from different origins and varieties in the chemical composition space. Meanwhile, Spearman’s rank correlation coefficients were calculated using R software to investigate the associations between chemical components and sensory/burning indicators, including sweetness and moistness, ash cohesion, burnability, cleanliness, and delicacy.

**Results:**

The PCA score plots revealed relatively independent clusters for samples from Sichuan and Hainan Tunchang, while samples from some origins (e.g., Hainan Danzhou, Hubei Yichang, and Hubei Danjiangkou) were positioned close to each other, suggesting certain chemical similarities. Spearman correlation analysis identified several chemical components (e.g., esters, phenols, and alkaloids) that showed strong correlations with specific sensory attributes. Among these, four candidate chemical markers—methyl stearate, pyridinylpyrrolone, 2,3’-bipyridine, and 2-(3-pyridyl)-2-pyrroline—met the predefined criteria for further investigation.

**Discussion:**

It should be emphasized that, due to the limited sample size (27 samples, with only 3 samples per origin), the PCA results are exploratory in nature and do not support definitive conclusions regarding origin discrimination. The correlation findings are hypothesis-generating and require independent validation with larger sample sets. This study provides preliminary data and candidate chemical targets for future research on cigar tobacco leaf quality with expanded sample sizes and validated analytical methods.

## 1 Introduction

The characteristics of tobacco leaves are a critical factor influencing the quality of cigar tobacco, and the chemical composition of tobacco leaves, the accuracy of origin traceability, and sensory evaluation collectively constitute the key elements of cigar quality control (Li et al., 2024a; Shi et al., 2004). The chemical composition of tobacco leaves not only directly reflects their growing environment and genetic characteristics but also serves as the material basis for sensory quality. A systematic analysis of these chemical components is a prerequisite for achieving precise quality control of cigars (Xiong et al., 2025). In recent years, researchers have employed various detection methods to objectively differentiate tobacco products. For instance, Zhang et al. (2022) identified 125 chemical components using solid-supported liquid-liquid extraction coupled with gas chromatography-mass spectrometry and chemometric analysis, establishing an OPLS-DA model to successfully distinguish tobacco extracts from Yunnan, Zimbabwe, and other origins. Pan Xi et al. proposed a method based on near-infrared spectroscopy (NIRS) combined with supervised pattern recognition, providing an efficient, accurate, and non-destructive analytical technique for cigarette brand identification (Pan et al., 2020). Hu Jun et al. utilized static headspace gas chromatography-mass spectrometry (SHS-GC-MS/MS) and gas chromatography-ion mobility spectrometry (GC-IMS), combined with principal component analysis (PCA) and partial least squares-discriminant analysis (PLS-DA), to comparatively analyze key aroma components in two types of cigar tobacco leaves (Hu et al., 2023). Collectively, these studies have demonstrated the value of instrumental analysis combined with multivariate statistics for tobacco differentiation. However, several gaps remain. First, most of these investigations have relied on a single analytical platform or a limited set of chemical features, which may not adequately represent the full compositional complexity of cigar tobacco leaves. Second, the integration of multi-source chemical data with sensory quality assessment—an essential step for understanding the chemical basis of consumer perception—has been rarely addressed in a systematic manner. Third, the potential of such integrated approaches for origin traceability and varietal discrimination in cigar tobacco remains largely unexplored, particularly in the context of domestic Chinese cultivars.

The chemical diversity of tobacco constituents renders a single analytical platform insufficient for comprehensive coverage. Different chemical components exhibit marked differences in physicochemical properties such as polarity, volatility, thermal stability, and molecular weight, necessitating complementary analytical strategies for the systematic characterization of the tobacco chemical profile. Gas chromatography-mass spectrometry (GC-MS/MS) is well-suited for the separation and identification of volatile and semi-volatile, thermally stable, and low-polarity compounds, with access to standard mass spectral databases (e.g., the NIST library) enabling reliable putative matching of unknown volatiles. Liquid chromatography-mass spectrometry (LC-MS/MS), in contrast, excels in the detection of thermally labile, highly polar, or moderately polar compounds, and is particularly suitable for the sensitive quantitative analysis of characteristic small molecules such as alkaloids and nitrogen-containing heterocycles. The combined use of these two mass spectrometric platforms has been widely adopted in metabolomics research, leveraging their complementary strengths in compound coverage to achieve a more comprehensive characterization of sample chemical composition (Bharat et al., 2025; Zheng et al., 2019). Furthermore, macromolecular nutrients in tobacco leaves—such as starch and protein—also exert significant influences on sensory quality: starch content is closely related to the aroma quality and combustion characteristics of tobacco leaves, while protein may decompose during combustion to generate nitrogen-containing compounds that affect smoke irritancy and taste (Chen et al., 2026; Zhang et al., 2006). However, these two classes of macromolecular substances are difficult to analyze directly by GC-MS/MS or LC-MS/MS in a high-throughput manner, and thus require specific detection via biochemical assay kits.

Ultimately, the market competitiveness of cigars depends on consumer sensory experience, and the intrinsic correlation between chemical composition and sensory evaluation indicators represents a key pathway for achieving targeted quality optimization (Xiong et al., 2025). Existing research primarily focuses on simple correlation analyses between individual chemical components and specific sensory indicators, lacking in-depth exploration of the nonlinear, multi-dimensional association mechanisms between complex chemical systems and comprehensive sensory performance. This gap results in process optimization lacking clear, targeted guidance, making it difficult to achieve precise alignment between chemical composition regulation and sensory quality improvement.

Based on the above-mentioned considerations, this study constructs a multi-dimensional chemical analysis framework of “GC-MS untargeted screening + LC-MS targeted quantification + biochemical kit-assisted detection”. Centering on the core demand for quality control of cigar tobacco leaves, a systematic research workflow of “multi-source chemical characteristic analysis—feature extraction—sensory quality correlation” is established. Firstly, technologies including gas chromatography-mass spectrometry (GC-MS), liquid chromatography-mass spectrometry (LC-MS) and biochemical kits are adopted to conduct multi-dimensional chemical composition analysis on cigar tobacco leaves from different producing areas and varieties, so as to build a comprehensive chemical characteristic database. Secondly, to address the redundancy of high-dimensional data, principal component analysis (PCA) is applied for core feature extraction and dimensionality reduction, revealing the natural distribution characteristics and clustering trends of tobacco leaves of different producing areas and varieties in the chemical composition space. Finally, the correlation mechanism between chemical components and sensory evaluation indicators (sweetness-smoothness, ash cohesion, combustibility, cleanliness, fineness, etc.) is deeply explored, and key chemical targets related to sensory quality are screened via Spearman correlation analysis. This study aims to provide theoretical support and technical references for raw material screening, origin traceability, variety identification and targeted quality optimization of cigar tobacco leaves, and facilitate the full-chain improvement of the cigar industry ranging from raw material control to quality enhancement.

## 2 Materials and methods

### 2.1 Materials, reagents, and instruments

The tobacco leaf samples involved in the experiment were obtained from China Tobacco Sichuan Industrial Co., Ltd., comprising 27 samples from 9 different origins and 3 different varieties. Detailed information is provided in Supplementary Table S1. The alkaloid standards used for quantification and other reagents are listed in Supplementary Table S2, and the main instruments and equipment are shown in Supplementary Table S3.

### 2.2 Experimental methods

#### 2.2.1 GC-MS/MS analysis conditions

This study adopted an untargeted profiling analysis strategy. After samples were extracted with methanol, data acquisition was performed in the GC-MS Full Scan mode to conduct unbiased detection of all volatile chemical components in the samples. Following peak detection and deconvolution of the raw data, compound identification was carried out via the NIST 17 mass spectral library search, and semi-quantitative analysis was performed using the internal standard method. The final data were applied for multivariate statistical analysis.

Sample pretreatment: Accurately weigh 20 mg of each of the 27 tobacco samples, add 2 mL of chromatographic grade methanol, shake thoroughly, and extract at room temperature for 24 h. Subsequently, filter through a 0.22 μm membrane into a 2 mL centrifuge tube, and store at 4 °C.

Chromatographic conditions: The chromatographic column used was an Rtx-WAX column. The initial temperature was 50 °C, held for 2 min, then increased to 280 °C at a rate of 5 °C/min and held for 10 min, followed by an increase to 310 °C at a rate of 30 °C/min, held for 2 min. The carrier gas was helium, with a septum purge flow of 3.0 mL/min. The injector temperature was 290 °C, and the pressure was 9.7853 psi. The injection volume was 1 μL with a split ratio of 50:1. The total run time was 61 min.

Mass spectrometry conditions: The ion source was electron ionization (EI), with an ion source temperature of 230 °C and an electron energy of 70 eV. The scan mode was full scan, with a mass scan range (m/z) of 20–550, an acquisition rate of 5 spectra/s, and an acquisition time of 200 ms/spectrum. The run time was 10 min.

Compound Identification: The NIST 17 mass spectral library was used for spectral library search for qualitative analysis. A match score ≥70 was set as the threshold for preliminary identification, and comprehensive judgment was performed combined with chromatographic retention behavior. Referring to the metabolite identification confidence-level classification system, compounds were classified into three levels according to match scores: high-confidence tentative identification (Level 1) for match scores ≥90, medium-confidence tentative identification (Level 2) for match scores ranging from 80 to 89, and preliminary annotation (Level 3) for match scores ranging from 70 to 79. Compounds with match scores below 70 were excluded from subsequent statistical analysis.

It should be noted that compound identification in this study was solely based on NIST 17 mass spectral library searching. Retention index (RI) was not adopted for auxiliary verification, and authentic standards were not used for confirmation. Therefore, all compounds reported in this study represent tentative identification or putative annotation rather than confirmed identification. Follow-up studies will further improve identification reliability through standard-sample comparison and retention-index verification.

GC-MS/MS semi-quantitative analysis: Prior to injection, transfer 50 μL of the internal standard (methyl octanoate in methanol at a concentration of 0.050 μg/μL) and 950 μL of the tobacco sample into an injection vial. Volatile components were analyzed using a semi-quantitative method, assuming a correction factor of 1. The relative concentration of each volatile component was calculated based on the internal standard concentration, internal standard volume, internal standard peak area, and the peak area of the volatile component, using the following formula:

$$C=\frac{C_{IS}V_{IS}A}{A_{IS}V}$$

where: C is the volume concentration of the volatile component (μg/mL); $C_{IS}$ is the volume concentration of the internal standard (μg/μL); V_IS_ is the volume of the internal standard (μL); $A_{IS}$ is the peak area of the internal standard; A is the peak area of the volatile component; V is the constant volume of the sample. It should be noted that since the correction factor for all compounds is assumed to be 1 and individual standard curves for each compound are not adopted, the concentrations reported in this study are relative contents, which are only used for horizontal comparison among samples and do not represent the absolute true content of each component.

Full methodological validation was performed in this study. Methyl n-caprylate was selected as the internal standard with a stock solution concentration of 50 μg/mL. The calibration curve was established by plotting the peak area ratio of analyte to internal standard against analyte concentration. Good linearity was obtained within the tested concentration range, with all coefficients of determination (R^2^) higher than 0.990. The signal-to-noise ratio method was adopted to determine the Instrument Detection Limit (IDL) and Instrument Quantification Limit (IQL): the IDL was 0.0004 μg/mL (S/N = 3), and the IQL was 0.0025 μg/mL (S/N = 10).

Precision calculation (intra-day repeatability): Take the methyl n-octanoate standard working solution with a concentration of 5 μg/mL, and perform 6 consecutive parallel injections. Examine the relative standard deviation (RSD) of retention time and peak area. The results show that the RSD of methyl n-octanoate retention time is less than 0.5%, and the RSD of peak area is less than 3.0%. The instrument operates with good stability and meets the requirements for batch sample testing.

#### 2.2.2 LC-MS/MS analysis conditions

##### 2.2.2.1 Alkaloid analysis conditions

Sample pretreatment: Accurately weigh 20 mg of each of the 27 tobacco samples, add 2 mL of chromatographic grade methanol, shake thoroughly, and extract at room temperature for 24 h. Subsequently, filter through a 0.22 μm membrane into a 2 mL centrifuge tube, and store at 4 °C.

Chromatographic conditions: The chromatographic column used was a Shim-pack Velox column (100 mm length, 2.1 mm inner diameter, 1.8 μm particle size). The column temperature was maintained at 30 °C. Mobile phase A consisted of water containing 0.1% formic acid, and mobile phase B was acetonitrile. The gradient elution program was as follows: 0–3.5 min, 95% A; 3.5–3.6 min, 95%–10% A; 3.6–4.6 min, 10%–10% A; 4.6–4.7 min, 10%–95% A; 4.7–5.5 min, 95%–95% A. The flow rate was 0.30 mL/min, and the injection volume was 10 μL.

Mass spectrometry conditions: A heated electrospray ionization (H-ESI) source was used, and scanning was performed in both positive and negative ion modes. The ion transfer tube temperature was set to 325 °C, and the evaporator temperature was 350 °C. The spray voltage was +3500 V/2000 V in negative mode. The nebulizer gas pressure was 35 psi, the sweep gas pressure was 0 psi, and the auxiliary gas pressure was 5 psi. The mass scan range (m/z) was 2–2000. Basis for scan mode selection: In this study, Q3 full-scan (Q3 MS) and product ion scan modes of triple-quadrupole mass spectrometry were adopted for data acquisition. The above-mentioned modes were selected instead of the multiple reaction monitoring (MRM) mode mainly based on the following considerations: (1) This study aims to perform untargeted screening and semi-quantitative comparison of as many nitrogen-containing compounds (alkaloids, amino acids, nitrogen-containing heterocycles, etc.) in tobacco leaves as possible, rather than targeted quantification of only a small number of known compounds; (2) Without comprehensive knowledge of alkaloids and nitrogen-containing compounds in each tobacco leaf sample, it is impossible to pre-determine the optimal precursor-to-product ion pairs and collision energies for all analytes. Q3 full-scan and product ion scan provide a “discover-first-verify-later” workflow; (3) Q3 full-scan can acquire full-scan MS^1^ spectra of all ionizable components in samples, and product ion scan can acquire full-scan MS^2^ spectra of specific compounds. The combination of both provides abundant fragment information for compound structure identification.

It should be noted that the MRM mode exhibits remarkable advantages in sensitivity and selectivity and serves as the gold-standard method for targeted quantitative analysis. The scan-mode selection in this study is based on the research orientation of untargeted screening, rather than negating the advantages of the MRM method. In subsequent verification studies, dedicated MRM methods will be established for key alkaloids (such as nicotine, nornicotine, anatabine, etc.) to achieve accurate quantification with higher sensitivity and selectivity.

Standard Curve and Quantification Method: Accurately weigh five reference standards including 1,2,4-triazole, indole, nornicotine, 1,2,3,6-tetrahydro-2,3-dipyridyl (anatabine) and anabasine respectively. Dissolve them in chromatographic-grade methanol and perform serial dilution to prepare a series of mixed standard working solutions at 12 concentration levels: 0.00025, 0.00050, 0.00100, 0.00250, 0.00500, 0.01000, 0.02500, 0.05000, 0.10000, 0.25000, 0.50000 and 1.00000 μg/mL. Take the concentration X (ppb) of each standard as the abscissa and the corresponding chromatographic peak area Y as the ordinate. Conduct linear regression fitting via the least-squares method (weighting factor: 1/X, no forced zero-point intercept) to obtain the standard curve equations for each compound (Table 1). The results show that 1,2,4-triazole, nornicotine and anabasine exhibit favorable linear relationships within the range of 0.25–1000.00 ppb (R^2^ > 0.995); the linearity of 1,2,3,6-tetrahydro-2,3-dipyridyl is acceptable (R^2^ = 0.9812); indole presents a relatively low coefficient of determination (R^2^ = 0.8263), which may be related to the stability of its ionization efficiency under the present chromatographic conditions. The quantification accuracy can be further improved subsequently by optimizing mass spectrometry parameters or adopting an internal standard calibration strategy.

**TABLE 1:** Standard curve equations and coefficients of determination for the five target compounds.

Compound	Standard curve equation	R^2^
1,2,4-Triazole	Y = 21485.6 + 45070.9·X	0.9952
Indole	Y = 87470.4 + 3210.98·X	0.8263
Nornicotine	Y = 544041 + 395480·X	0.9953
1,2,3,6-Tetrahydro-2,3'-bipyridine (Anatabine)	Y = 3.43149 × 10^6^ + 257399·X	0.9812
Anabasine	Y = 1.25378 × 10^6^ + 197880·X	0.9953

Y represents the peak area, X represents the concentration of the compound (ppb). Linear regression was performed using a 1/X weighting factor without forcing the origin.

The instrumental detection limit (IDL) and instrumental quantification limit (IQL) were calculated using the linear interpolation method based on the measured signal-to-noise ratios (S/N) of the serially diluted standard solutions. The IDL was determined at S/N = 3, and the IQL was determined at S/N = 10. The results showed that the IDL and IQL for all five target compounds were 0.0047 μg/mL and 0.00607 μg/mL, respectively. The concentrations of target analytes in all tobacco samples exceeded the IQL, falling within the effective quantitative range. These IDL and IQL data indicate that the Q3 full-scan mode provides sufficient sensitivity for the semi-quantitative analysis of nitrogen-containing compounds in tobacco samples in this study. All alkaloid concentrations in the tested samples were above 6.07 ppb, within the effective quantitative range of 10.00–1000.00 ppb.

Precision and accuracy: Mixed standard solutions at three concentration levels (0.5, 25, and 50 ppb) were selected to evaluate intra-day precision (six consecutive injections) and inter-day precision (three consecutive days, three injections per day). The results showed that the RSD values for peak areas were ≤4.0% and those for concentrations were ≤5.0% for all compounds, indicating good instrumental precision. Accuracy was evaluated using the standard addition method. Low, medium, and high levels of the five mixed standards were spiked into tobacco samples with known contents, and the recoveries were calculated following the same sample preparation procedure. The recoveries for all compounds ranged from 88.0% to 102.0%, with RSD ≤4.5%, confirming that the method accuracy met the requirements for quantitative analysis.

Control of instrument drift and signal fluctuation: All samples were analyzed consecutively within a single analytical batch. In the sample sequence, a quality control (QC) sample—prepared by mixing equal aliquots of all test samples—was inserted after every 6–8 samples to monitor the drift trend of the instrument response. Prior to daily analysis, the instrument was fully warmed up and the mobile phase was equilibrated for no less than 30 min; sample acquisition commenced only after system pressure, baseline, and mass spectrometric response had stabilized. The intra-day precision RSD≤4.0% obtained from method validation confirmed the stability of the instrument within a single analytical batch.

Sample determination: For actual sample measurement, each sample was injected once. To monitor the stability of the analytical sequence, a quality control standard (mixed standard solution at 25 ppb) was injected after every 10 samples. The deviation of all QC measurements from the theoretical values was within ±15%, indicating stable instrument response throughout the entire analytical sequence. The repeatability and precision of the method were confirmed by the replicate injections of standards and the standard addition recovery experiments described above, supporting the reliability of single-injection measurements for cross-sample comparisons.

Assessment of variability: Each tobacco sample was analyzed with a single injection in this study; therefore, standard deviation (SD) could not be provided for individual samples. The precision of the method was verified through replicate standard experiments: intra-day precision (six consecutive injections) yielded RSD ≤4.0% for peak areas and ≤5.0% for concentrations; inter-day precision (three consecutive days, three injections per day) yielded RSD ≤5.0% for concentrations; the standard addition recovery experiments yielded RSD ≤4.5% (n = 6). To characterize inter-sample variability, the range, median, mean, and coefficient of variation (CV) for each compound across the 27 samples are reported, and Kruskal-Wallis test followed by Dunn’s *post hoc* test was employed to evaluate the significance of differences among different origins.

##### 2.2.2.2 Amino acid analysis by HPLC with pre-column derivatization

Standard solution preparation: Aliquots of 40 μL of a 250 mmol/L amino acid mixed standard solution were transferred and diluted to various volumes with Milli-Q ultrapure water to obtain standard solutions at different concentration levels.

Derivatization of standards: A 10 μL aliquot of 400 μmol/L amino acid standard solution was placed into a 6 mm × 50 mm sample vial bottom, followed by the addition of 60 μL of borate buffer solution. The mixture was vortex-mixed for 10 s, after which 10 μL of AQC derivatization reagent solution was added under continuous vortexing. The vial was sealed with parafilm, allowed to stand at room temperature for 1 min, and then heated at 55 °C for 20 min. After cooling to room temperature, a 10 μL aliquot was injected for HPLC analysis.

Extraction of amino acids from tobacco samples: Accurately weighed 1.0 g of tobacco sample was placed in a 50 mL centrifuge tube, and 10 mL of 20 mmol/L hydrochloric acid solution was added. The mixture was extracted by ultrasonication or shaking for 2 h. After extraction, the tube was shaken thoroughly and allowed to stand for settling.

Derivatization of sample extracts: A 30 μL aliquot of the supernatant was transferred into a chromatographic vial equipped with an insert. Then, 60 μL of borate buffer was added and mixed rapidly. Under vortexing, 10 μL of AccQ·Tag amino acid derivatization reagent was introduced, and the mixture was vortexed for an additional 10 s. The vial was sealed with parafilm, left at room temperature for 1 min, and then placed in a 55 °C oven for derivatization for 10 min. After cooling to room temperature, the derivatized sample was ready for injection and detection at 250 nm.

Chromatographic conditions: The separation was performed on an AccQ·Tag amino acid analysis column (150 mm × 4.6 mm, 5 μm particle size, Waters) maintained at 35 °C. The injection volume was 25 μL. Mobile phase A consisted of Waters mobile phase A diluted 10-fold with ultrapure water, and mobile phase B consisted of 60% aqueous acetonitrile. The flow rate was 1.0 mL/min. Detection was carried out at wavelengths of 250 nm and 440 nm. The gradient elution program is presented in Table 2. Method Validation Parameters of LC-MS/MS for Seven Amino Acid Compounds in Table 3.

**TABLE 2:** Gradient elution conditions.

Time	A	B
0	100	0
0.5	98	2
15	93	7
19	90	10
32	67	33
33	67	33
34	0	100
37	0	100
38	100	0
45	100	0

**TABLE 3:** Method validation parameters of LC-MS/MS for seven amino acid compounds.

Number	Compound	Regression equation	Linear range	R^2^	Precision RSD (%)	Repeatability RSD (%)	IQL (μg/mL)
1	Asp	Y = 389.3482X	0.1–200	0.9997	0.23	1.21	0.01
2	Ser	Y = 33.14908X	0.1–200	0.9947	0.65	1.36	0.01
3	Glu	Y = 5.8852X	0.1–200	0.9914	0.38	1.52	0.01
8	Ala	Y = 2.0577X	0.1–200	0.9984	0.42	1.69	0.01
9	Pro	Y = 1.6330X	0.1–200	0.9981	0.48	1.78	0.01
11	Asn	Y = 3.7759X	0.1–200	0.998	0.82	1.24	0.01
12	Trp	Y = 12.1817X	0.1–200	0.9981	0.91	1.36	0.01

#### 2.2.3 Total starch detection method

Sample pretreatment: Accurately weigh 10 mg of fresh sample, add 1 mL of Reagent 1, mix thoroughly, and transfer to an EP tube. Extract in an 80 °C water bath for 30 min, then centrifuge at 10,000 r/min for 10 min. Discard the supernatant and retain the precipitate. Add 0.5 mL of distilled water to the precipitate and gelatinize in a 95 °C water bath for 15 min. After cooling, add 0.35 mL of Reagent 2 and extract at 25 °C for 15 min, shaking 3–5 times. Add 0.85 mL of distilled water, mix well, and centrifuge at 10,000 r/min for 10 min. Collect the supernatant as the sample solution. For the working solution, add 7.5 mL of distilled water to Reagent 3, then slowly add 42.5 mL of concentrated sulfuric acid while stirring continuously until fully dissolved.

Total starch detection: Transfer 0.2 mL of the sample solution and 1 mL of the working solution to an EP tube, heat in a 95 °C water bath for 10 min, cool to room temperature, and measure the absorbance (A) at a wavelength of 620 nm. The calculation method is as follows:

Starch content (mg/g fresh weight) = 0.289 × (A + 0.0295) ÷ W, W is the sample mass (g).

#### 2.2.4 Total protein detection method

Sample pretreatment: Accurately weigh 10 mg of fresh sample, add 900 μL of PBS buffer, homogenize in an ice bath, and centrifuge at 10,000 r/min for 10 min. Collect 50 μL of the supernatant for analysis.

Total protein detection: Pipette 40 μL of the supernatant and mix separately with 2 mL of biuret blank reagent and 2 mL of Doumas biuret reagent. Incubate at 25 °C for 30 min. Using a calibrated precision spectrophotometer with a cuvette light path of 1 cm, measure the absorbance at a wavelength of 540 nm. When measuring the absorbance of the sample tube and the reagent blank tube, zero the instrument with a distilled water blank tube. When measuring the absorbance of the sample blank tube, zero the instrument with a biuret reagent blank tube. The calculation method is as follows:

Total protein (g/L) = (Ac/0.298) × 51, Corrected absorbance (Ac) = At - (Ar + As); At is the absorbance of the sample tube; Ar is the absorbance of the reagent blank tube; As is the absorbance of the sample blank tube; 0.298 is the specific absorption coefficient of the protein–biuret complex.

#### 2.2.5 Sensory evaluation

The tobacco leaves were rolled into cigarettes with a ring gauge of 38 and a length of 110 mm. The cigarettes were placed in a constant temperature and humidity chamber at 20 °C and a relative humidity of 65% to equilibrate moisture for 30 days. Seven panelists qualified in sensory evaluation were organized to conduct the evaluation according to the requirements of the sensory evaluation form (Table 4). The sensory quality evaluation of the tobacco leaves primarily focused on three aspects: aroma note characteristics, off-flavors, and quality characteristics. All sensory evaluators are in-house researchers who have received systematic training and obtained professional sensory evaluation qualifications. All of them conduct unified sensory smoking assessments and finalize the scores after group discussion.

**TABLE 4:** Evaluation table of sensory quality and style of domestic cigar tobacco leaves.

Sample:		Type:					Origin:	Variety:	Grade:		Year:		
Style Characteristics	□ Significant □ Relatively Significant □ Moderate □ Relatively Weak □ Weak						Concentration	□ Full □ Relatively Full □ Moderate □ Relatively Light □ Light □					
Attribute	Descriptor	5	4	3	2	1	Attribute	Descriptor	5	4	3	2	1
		Significant	Relatively Significant	Moderate	Relatively Weak	Weak			Heavy	Relatively Heavy	Moderate	Relatively Light	Light
Aroma Notes	Nutty	​					Off-Flavors	Proteinaceous		​			
	Beany	​						Earthy		​			
	Coffee	​						Green		​			
	Cocoa	​						Scorched/Burnt		​			
	Woody	​						Floral		​			
	Spicy	​						Woody		​			
	Fruity	​						Regional		​			
	Fresh Sweet	​						Other		​			
	Caramelized Sweet	​					Attribute	Descripto		9	6	3	
	Honey Sweet	​								Good	Moderate	Poor	
	Floral	​					Quality Characteristics	Aroma	Aroma Quantity	​			
	Smoky	​							Richness	​			
	Medicinal	​							Maturity	​			
	Milky	​						Smoke	Harshness	​			
	Resinous	​							Smoothness	​			
	Roasted	​							Delicacy	​			
	Hay-like	​						Taste	Sweetness	​			
	Leathery	​							Cleanliness	​			
	Earthy	​							Aftertaste	​			
​	Other	​					​	Combustion	Combustibility	​			
									Ash Color	​			
									Ash Cohesiveness	​			
Aroma-Oriented□ Taste-Oriented□ Filler□ Supplementary□													
Overall Evaluation:													
Evaluator: Date:													

### 2.3 Data Processing

To clarify the differences in the mass fractions and compositions of the 27 tobacco samples, Microsoft Excel 2024 was used for data statistics. Principal component analysis (PCA) were performed using SPSS Statistics 27.0.1 and Graphpad Prism 10.1.2. To elucidate the relationship between chemical components and sensory quality, correlation analysis was conducted using R language to calculate correlation coefficients between the relative contents of chemical components and the sensory evaluation results, and a heatmap was generated accordingly.

Screening criteria for candidate chemical markers: This study strictly distinguishes between “compounds significantly correlated with sensory indicators” and “compounds that can be regarded as candidate chemical markers”. In Spearman correlation analysis, compounds satisfying |r| > 0.5 and P < 0.05 are defined as “significantly correlated compounds”. Such compounds only suggest a correlative trend with specific sensory indicators at the statistical level. On this basis, the screening of putative chemical markers must satisfy all of the following criteria simultaneously: (1) |r| > 0.6 and P < 0.01 to ensure correlation strength and statistical robustness; (2) the compound exhibits detectable concentration differences in samples from at least one origin or variety (coefficient of variation CV > 30%) to guarantee its practical discriminatory capacity; (3) the correlation is biologically or chemically reasonable, meaning the known functions, metabolic pathways or physicochemical properties of the compound can provide mechanistic explanations for its association with sensory indicators; (4) the correlation is supported by literature in similar studies or can be validated by consistent trends in independent data subsets of this study (e.g., among different varieties). Only compounds that meet all the above four criteria can be defined as “candidate chemical markers”. Significantly correlated compounds that fail to meet the marker criteria are reported merely as “notable correlations” in the paper, without causal or marker-level inferences.

## 3 Results and discussion

### 3.1 Analysis of chemical components in cigar tobacco leaves

#### 3.1.1 GC-MS/MS results of different tobacco samples

Twenty-seven tobacco leaf samples were analyzed by GC-MS, and a total of 101 compounds were screened (Supplementary Table S4). Among them, 50 volatile chemical constituents were subjected to semi-quantitative analysis. These compounds could be classified into seven categories including alkaloids, alcohols, esters, and ketones based on their structural characteristics and biosynthetic pathways. Based on their structural characteristics and biosynthetic pathways, these compounds were classified into seven major categories, including alkaloids, alcohols, esters, and ketones. It should be noted that, as the GC-MS/MS semi-quantitative analysis did not establish independent calibration curves for all detected compounds, the obtained values represent relative quantitative results and are intended solely for cross-sample comparisons, rather than for precise absolute quantification. Detailed concentration data for all chemical substances across the three varieties and nine origins are provided in Supplementary Material Supplementary Tables S5-S7. A heatmap was constructed based on the total content of the seven chemical categories to visualize the overall chemical profiles of the samples (Figure 1). Alkaloids were found to be the dominant chemical class in the majority of samples. Alcohols were notably enriched in samples of Yunxue No. 2 and Dexue No. 4 from the DAZ and PE origins. An anomalously high abundance of alkanes was detected in Dexue No. 4 samples from the T origin. Clear differences in the accumulation patterns of various compound classes were observed among different varieties and origins.

**FIGURE 1:**
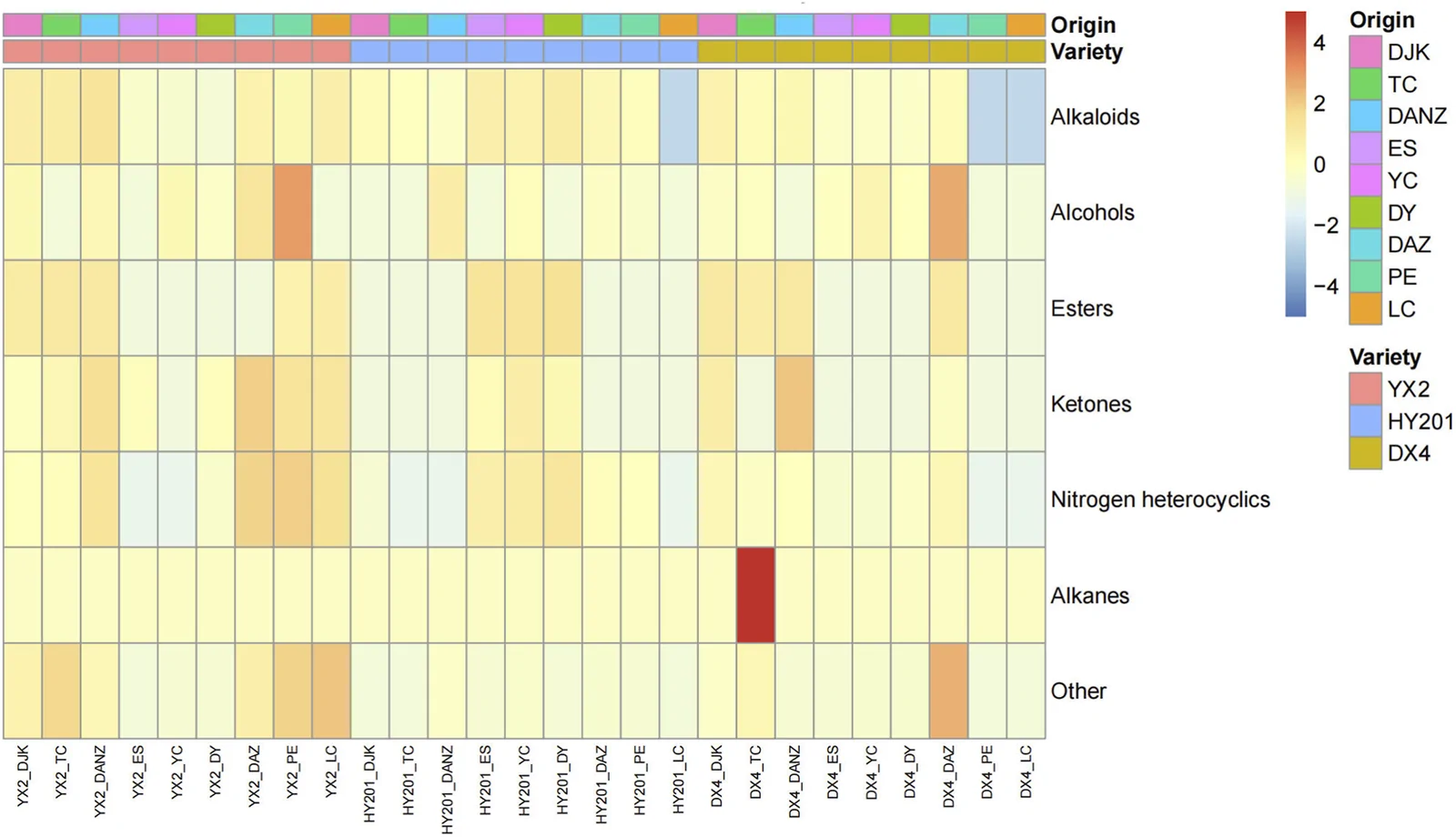
Heatmap of relative content of chemical substances.

#### 3.1.2 LC-MS/MS results of different tobacco samples

Through LC-MS/MS targeted quantitative analysis, the contents of selected alkaloids, nitrogen-containing heterocyclic compounds, and amino acids in the 27 tobacco samples were qualitatively and quantitatively determined (Tables 5 and 6). This approach transitions the characterization of tobacco signature components from broad-spectrum discovery via GC-MS/MS screening to precise quantification via LC-MS/MS, thereby establishing a chemometric foundation for subsequent multi-source data fusion analysis.

**TABLE 5:** Composition and content of alkaloids and nitrogen-containing heterocyclic substances in different tobacco samples (μg/mL).

Category	Alkaloids					Nitrogen heterocyclics				
Sample	Nornicotine	RSD (%)	Anatabine	RSD (%)	Anabasine	RSD (%)	Indole	RSD (%)	1,2,4-Triazole	RSD (%)
1	17.92	0.87	26.08	1.18	32.90	2.12	125.75	2.94	1.41	4.99
2	7.54	0.23	25.00	1.08	33.73	3.35	106.36	1.60	0.44	6.98
3	14.69	3.47	26.81	4.04	35.36	3.10	122.23	1.81	1.05	0.61
4	8.25	0.13	10.57	0.61	14.76	0.61	86.98	0.35	0.20	3.00
5	2.20	0.14	4.16	0.46	10.88	0.85	72.53	0.92	0.14	1.86
6	7.26	1.23	5.56	0.74	8.76	0.72	71.24	1.84	0.50	2.07
7	14.66	2.60	27.65	2.63	31.68	1.18	118.01	1.00	0.50	1.48
8	2.85	4.58	15.54	3.31	20.16	0.85	79.04	0.62	0.44	4.15
9	13.84	2.35	27.86	2.55	28.22	0.45	123.05	1.47	0.31	3.32
10	12.66	2.66	17.11	2.72	26.78	0.67	98.34	2.44	1.49	6.61
11	11.72	0.37	11.92	0.81	16.79	0.49	61.10	0.26	1.99	1.64
12	3.79	0.62	10.55	1.08	15.68	0.45	70.85	0.70	1.67	2.13
13	15.52	1.03	23.87	3.37	32.14	0.94	116.82	0.30	0.24	1.53
14	14.84	1.44	24.20	1.46	27.15	3.59	103.57	1.58	0.45	1.77
15	19.09	3.32	23.74	4.85	36.58	2.03	121.23	2.15	0.49	0.76
16	21.27	1.79	30.65	1.91	35.53	2.62	131.12	3.67	0.58	3.10
17	10.93	1.67	36.76	2.15	32.98	1.93	122.16	1.23	0.24	5.05
18	14.33	2.50	32.85	2.78	33.61	0.17	125.48	1.28	0.97	1.41
19	12.29	2.31	28.79	2.18	23.25	0.66	94.02	0.95	4.47	2.71
20	7.28	0.31	18.07	0.24	18.59	0.71	83.73	1.10	5.91	2.20
21	9.72	1.54	21.36	1.96	19.74	0.26	94.07	2.15	5.40	3.71
22	11.83	1.42	16.64	1.71	17.74	0.93	94.59	0.36	0.44	11.29
23	3.79	2.68	10.41	4.36	15.39	0.39	76.42	1.23	0.49	0.35
24	5.11	2.33	11.45	3.38	13.20	1.17	72.50	0.97	0.50	0.50
25	11.13	1.84	12.73	2.56	19.70	0.64	92.21	1.83	0.49	0.27
26	3.79	2.40	9.31	6.56	14.54	2.48	67.23	3.06	0.45	2.09
27	6.02	0.94	11.28	5.10	16.29	0.58	73.03	0.58	0.13	4.10

The RSD values in the table refer to the relative standard deviations between the measured values and fitted values of each concentration point during the standard-curve fitting for each compound. They are derived from the regression statistics output of the instrument software and do not represent the variability of repeated sample determinations.

**TABLE 6:** Composition and content of amino acids in different tobacco samples.

Sample	Ser	RSD (%)	Pro	RSD (%)
1	9.873	0.81	0.462	0.25
2	10.200	0.78	0.274	0.42
3	9.971	1.34	0.376	0.31
4	10.432	0.48	0.140	0.82
5	10.074	0.70	0.133	1.25
6	10.562	0.76	0.131	0.88
7	9.374	0.85	0.139	0.83
8	10.497	0.68	0.136	0.85
9	9.438	0.85	0.247	0.57
10	10.259	0.78	0.844	0.14
11	9.870	0.81	0.966	0.12
12	10.637	1.13	0.887	0.40
13	8.925	0.90	0.267	2.06
14	9.360	0.53	0.166	3.32
15	8.794	1.03	0.133	0.87
16	9.941	0.80	0.619	0.19
17	9.344	0.86	0.275	1.83
18	10.545	0.86	0.787	0.64
19	10.216	0.88	1.846	0.14
20	10.997	0.73	2.054	0.16
21	9.966	1.01	1.951	0.16
22	10.329	0.97	0.186	0.62
23	11.055	0.91	0.146	0.79
24	10.832	0.74	0.146	0.79
25	9.254	0.50	0.168	0.99
26	9.228	0.87	0.153	1.15
27	9.432	0.85	0.281	1.41

Contents are expressed in μg/mL (based on 1.0 g sample/10 mL extract). RSD: relative standard deviation (%), derived from calibration curve fitting parameters or replicate measurements.

Alkaloids are a class of nitrogen-containing heterocyclic compounds widely found in nature, exhibiting certain biological and pharmaceutical activities (Richiro and Ikuro, 2023). Generally, nicotine has the highest content, accounting for approximately 90% of the total alkaloid composition, while the remaining alkaloids, such as nornicotine, anatabine, and anabasine, typically account for about 10% of the total content (Lin et al., 2021). Anabasine shows a negative correlation with strength scores and has an adverse effect on aroma, with relatively low content in tobacco (Gunasekaran M et al., 2020). The content of nornicotine varies, possibly due to its instability and susceptibility to oxidation, acylation, and nitrosation reactions; the resulting substances may negatively affect the aroma of cigar tobacco (Dang et al., 2023), including myosmine and acylated nornicotine derivatives (Shi et al., 2023). Anatabine influences aroma quality, aroma quantity, irritation, and tobacco-specific nitrosamines (TSNAs) content (Zhou et al., 2020). Indole is an aromatic heterocyclic compound formed by the fusion of a benzene ring and a pyrrole ring (Chen H. et al., 2024). Indole alkaloids include indoles, monoterpenoid indoles, and bisindoles (Zhou et al., 2024). The sensory impact of indole on tobacco quality is concentration-dependent and exhibits a dual nature (Chi et al., 2019). At very low concentrations, indole imparts subtle floral and animal-like notes; however, as the concentration increases, its olfactory character shifts from floral to fecal-like odors (Wu et al., 2026). Previous studies have identified indole as a negative factor influencing sensory evaluation results, showing significant negative correlations with aroma quantity, aroma quality, and taste of tobacco leaves (Yu et al., 2006). Moreover, indole contents vary considerably among tobacco leaves from different production regions, which may contribute to the regional differences in sensory quality characteristics (Jiang et al., 2024).

Among the five target compounds, indole exhibited the highest mean content (613.73 μg/mL), while 1,2,4-triazole showed the lowest (4.06 μg/mL). Notably, 1,2,4-triazole displayed the largest coefficient of variation (CV = 131.5%) across the 27 samples, indicating that its content was most significantly influenced by origin and/or variety (Table 7). Anabasine, nornicotine, and 1,2,3,6-tetrahydro-2,3′-bipyridine (anatabine) also exhibited considerable inter-sample variations, with CV values of 39.7%, 53.6%, and 57.1%, respectively. In contrast, indole showed a relatively low CV of 21.3%, suggesting comparatively stable accumulation across samples.

**TABLE 7:** Statistical summary of the contents of five LC-MS/MS target compounds across 27 samples.

Compound	Min	Max	Median	Mean	CV (%)
1,2,4-Triazole	0.05	73.46	1.56	4.06	131.5
Indole	336.50	847.33	613.73	613.73	21.3
Nornicotine	9.75	164.87	73.43	71.33	53.6
1,2,3,6-Tetrahydro-2,3′-bipyridine	6.01	232.54	84.84	106.02	57.1
Anabasine	645.09	2853.11	1494.15	1712.72	39.7

Kruskal-Wallis tests were performed to evaluate differences in the contents of the five target compounds among the nine origins. The results revealed significant differences across origins for 1,2,4-triazole (H = 18.52, P = 0.018), nornicotine (H = 17.63, P = 0.024), 1,2,3,6-tetrahydro-2,3′-bipyridine (anatabine) (H = 22.12, P = 0.005), and anabasine (H = 16.95, P = 0.031) at P < 0.05, whereas indole did not reach statistical significance (H = 13.86, P = 0.085). Subsequent Dunn’s *post hoc* tests further indicated that the 1,2,4-triazole content in samples from DAZ was significantly higher than that in samples from TC (P = 0.003) and LC (P = 0.008). The contents of nornicotine and anabasine in samples from DY were significantly higher than those from TC (P = 0.006 and P = 0.011, respectively). No significant differences in alkaloid contents were observed between YC and DANZ (P > 0.05), which is consistent with the close proximity of samples from these two origins in the PCA score plot.

Anabasine content ranged from 645.09 to 2853.11 μg/mL, with the highest value detected in sample 16 (Yunxue No. 2 from DY) and the lowest in sample 6 (Dexue No. 4 from TC), representing an approximately 4.4-fold difference. Nornicotine content ranged from 9.75 to 164.87 μg/mL, with the maximum in sample 15 (Dexue No. 4 from YC) and the minimum in sample 5 (Haiyan 201 from TC). The content of 1,2,3,6-tetrahydro-2,3′-bipyridine (anatabine) ranged from 6.01 to 232.54 μg/mL, with sample 17 (Haiyan 201 from DY) showing the highest value and sample 5 (Haiyan 201 from TC) the lowest.

Indole content ranged from 336.50 to 847.33 μg/mL, with sample 3 (Dexue No. 4 from DJK) exhibiting the highest level and sample 11 (Haiyan 201 from ES) the lowest. The content of 1,2,4-triazole ranged from 0.05 to 73.46 μg/mL, with the maximum in sample 20 (Haiyan 201 from DAZ) and the minimum in sample 22 (Yunxue No. 2 from PE). Notably, the 1,2,4-triazole content in the three samples from DAZ (sample 19–21, ranging from 67.39 to 73.46 μg/mL) was significantly higher than that in all other samples (ranging from 0.05 to 30.73 μg/mL), and this origin-specific difference was statistically confirmed by Dunn’s *post hoc* test (P <0.01).

From a varietal perspective, Yunxue No. 2 exhibited relatively higher contents of indole and anabasine, while Haiyan 201 showed notably elevated levels of 1,2,4-triazole. In contrast, Dexue No. 4 displayed a relatively balanced distribution of all five target compounds.

Aspartic acid (Asp), glutamic acid (Glu), alanine (Ala), asparagine (Asn), and tryptophan (Trp) were detected at concentrations below 0.01 μg/mL in all samples after unit conversion, approaching or falling below the IQL. Consequently, the relative standard deviations (RSD) calculated for these compounds lack statistical significance due to the extremely low mean values. Therefore, these amino acids are not reported in the main table; their original converted data are provided in the Supplementary Material for reference. In contrast, serine (Ser) and proline (Pro) were consistently and reliably detected in all samples at concentrations far exceeding the IQL, and their RSD values are statistically meaningful. Accordingly, these two amino acids are reported in the main text.

Serine was the most abundant free amino acid detected in all tobacco samples, with concentrations ranging from 8.794 to 11.055 μg/mL, and exhibited relatively low inter-sample variability (CV = 8.1%), suggesting that serine accumulation is largely consistent across varieties and origins. This stable abundance may reflect its central role as a key intermediate in plant primary metabolism, particularly in photorespiration and amino acid biosynthesis.

In contrast, proline showed a much wider concentration range (0.131–2.054 μg/mL) and substantially higher inter-sample variability (CV = 83.9%), indicating that proline accumulation is more sensitive to genotype and/or environmental factors. Notably, samples from DAZ (Sample19-21) exhibited the highest proline levels (1.846–2.054 μg/mL), which were approximately 10–15 times higher than those from TC (Sample 4–6, 0.131–0.140 μg/mL). Such pronounced differences may be attributed to the well-documented role of proline as a compatible osmolyte that accumulates in response to drought, salinity, and other abiotic stresses. The higher proline content in Sichuan samples might therefore reflect the distinct climatic and soil conditions of this region, which could impose greater water or osmotic stress on tobacco plants compared to Hainan.

From a sensory perspective, free amino acids serve as important precursors for Maillard reaction products and Strecker aldehydes during tobacco curing and combustion, contributing to the formation of desirable flavor and aroma compounds (Hu et al., 2024). The consistently high serine levels across all samples suggest that this amino acid may serve as a stable reservoir for such reactions, whereas the origin- and variety-dependent variation in proline content could contribute to the regional differences in sensory profiles, as proline-derived products are known to influence caramel-like and nutty flavor notes (Wang et al., 2026, Zhang et al., 2025). However, the specific contributions of individual amino acids to the final sensory quality of cigar tobacco remain to be elucidated through targeted sensory recombination or model system studies.

Taken together, the distinct accumulation patterns of serine and proline across origins and varieties highlight the differential regulation of amino acid metabolism in tobacco leaves and suggest that proline, in particular, may serve as a sensitive metabolic indicator of regional environmental conditions. These findings provide preliminary chemical insights that may inform the selection of tobacco varieties and cultivation practices for targeted flavor quality optimization, although further investigation with larger sample sets is warranted to confirm the observed trends.

#### 3.1.3 Starch and protein detection results of different tobacco samples

Carbon-nitrogen metabolism (Wang et al., 2025), as a fundamental life process in plants, influences the utilization and output of photosynthesis as well as macromolecule synthesis. The coordination of carbon-nitrogen metabolism during the growth period of tobacco affects the accumulation of starch. When starch accumulation in tobacco decreases, the balance of intrinsic chemical components in tobacco leaves is affected, leading to reduced quality and yield. Starch content in tobacco leaves is also correlated with aroma quality and quantity (Chen et al., 2025). Protein in tobacco leaves is another important component affecting tobacco quality. Excessive protein content may make the leaf texture harder (Wang et al., 2023); upon combustion, proteins decompose to produce nitrogen-containing compounds (such as ammonia, pyridines, pyrazines, etc.), which may increase the harshness, alkalinity, and bitterness of the smoke (Yang et al., 2019). Therefore, in this section, the starch content in cigar tobacco leaves and the protein content in tobacco leaf extracts were determined using kit-based assays (Table 8).

**TABLE 8:** Starch and Sertein contents of different tobacco samples.

Sample	Starch content (μg/g)	Protein conten (%)
1	45.01	4.47
2	16.84	4.93
3	42.27	4.31
4	22.04	2.31
5	26.67	2.77
6	27.38	0.46
7	40.97	1.39
8	31.43	1.54
9	31.00	2.00
10	32.59	0.31
11	26.52	3.70
12	24.20	9.86
13	30.85	5.24
14	24.20	4.77
15	30.13	6.16
16	29.84	4.16
17	29.99	3.70
18	32.44	6.32
19	37.64	4.77
20	34.61	1.23
21	41.689	3.85
22	24.78	3.85
23	29.84	6.32
24	26.37	2.31
25	32.30	3.08
26	33.02	0.92
27	44.00	3.70

### 3.2 Exploration of sample chemical composition based on principal component analysis

#### 3.2.1 Data dimensionality reduction analysis

To visualize the overall chemical similarities and differences among cigar tobacco samples from different origins and varieties, the original chemical variables were systematically filtered prior to PCA. The initial dataset comprised 64 chemical variables (Supplementary Table S8), including 50 volatile components detected by GC-MS/MS non-targeted screening, 5 alkaloids/nitrogen heterocyclics quantified by LC-MS/MS, starch and total protein measured by biochemical assay kits, and free amino acids. To eliminate redundant information and ensure model stability, variables were filtered according to the criteria described in Section 2.3: (1) variables with detection rates below 70% (i.e., missing in >30% of samples) were excluded; (2) variables with relative standard deviation (RSD) below 5% across samples were removed; (3) for variable pairs with Pearson correlation coefficient |r| > 0.95, only the variable more strongly associated with tobacco quality was retained. This procedure yielded 16 representative chemical indicators for subsequent PCA.

Principal components with eigenvalues >1 were extracted. The first four principal components accounted for 79.71% of the total variance (Table 9), indicating that they adequately captured the core information of the 16 chemical variables. Since the number of principal components equals the number of input variables (16), the cumulative variance contribution reached 100.00% when all 16 components were extracted, meaning that the 16 principal components can fully reconstruct the variance information contained in the 16 selected chemical variables.

**TABLE 9:** Explanation of the total variance of PCA.

Component	Initial eigenvalues			Extraction sums of squared loadings		
	Total	Percentage of variance	Cumulative contribution rate%	Total	Percentage of variance	Accumulate %
1	6.469	40.432	40.432	6.469	40.432	40.432
2	3.526	22.039	62.471	3.526	22.039	62.471
3	1.586	9.913	72.384	1.586	9.913	72.384
4	1.172	7.327	79.711	1.172	7.327	79.711
5	0.930	5.814	85.525	​	​	​
6	0.617	3.858	89.383	​	​	​
7	0.568	3.553	92.936	​	​	​
8	0.335	2.091	95.028	​	​	​
9	0.279	1.742	96.770	​	​	​
10	0.174	1.087	97.857	​	​	​
11	0.122	0.763	98.620	​	​	​
12	0.084	0.524	99.144	​	​	​
13	0.063	0.394	99.539	​	​	​
14	0.048	0.301	99.839	​	​	​
15	0.019	0.120	99.959	​	​	​
16	0.007	0.041	100.000	​	​	​

The component matrix (Table 10) was used to interpret the physicochemical meaning of each principal component. PC1 (40.43% variance) showed high loadings for alkaloids, including nicotine (0.925), (S)-(−)-anatabine (0.902), anabasine (0.832), anatabine (0.807), and indole (0.792), indicating that this component primarily reflects differences in alkaloid composition—the most important chemical feature distinguishing samples. PC2 (22.04% variance) was characterized by sclareol (0.803) and neophytadiene (0.631), representing the distribution of alcohols and terpenoids. PC3 (9.91% variance) was dominated by 1,2,4-triazole (0.922), capturing information on nitrogen heterocyclic compounds. PC4 (7.33% variance) was associated with starch (0.595) and protein (−0.595), reflecting macromolecular nutrient content. Eigenvalues and variance contributions are detailed in Table 11.

**TABLE 10:** Component matrix.

Chemical substance	Component			
	1	2	3	4
Nicotine	0.925	−0.110	0.190	−0.044
Nornicotine	−0.251	−0.790	−0.336	0.098
(S)-(−)-Anatabine	0.902	0.171	0.065	−0.183
2,4-Di-tert-butylphenol	0.410	0.482	−0.177	0.314
Neophytadiene	0.514	0.631	0.068	−0.334
Indole	0.792	−0.511	−0.087	0.098
Nornicotine	0.737	−0.526	−0.056	0.096
Anatabine	0.807	−0.4465	0.069	0.082
Anabasine	0.832	−0.485	−0.026	0.009
1,2,4-Triazole	−0.047	0.034	0.922	0.250
2,3′-bipyridine	0.781	0.383	−0.230	0.171
Sclareol	0.022	0.803	−0.377	−0.004
Starch	0.452	0.343	0.239	0.595
Protein	0.161	−0.301	0.250	−0.595
Serine	−0.681	−0.058	0.407	0.052
Methyl palmitate	0.696	0.484	0.223	−0.299

**TABLE 11:** Sensory evaluation results.

No.	Aroma quantity	Richness	Maturity	Harshness	Smoothness	Delicacy	Sweetness	Cleanliness	Aftertaste	Ash color	Ash cohesiveness	Combustibility
1	6	6	6	6	5.5	6	6	6	6	7.5	7.5	7.5
2	5.5	5	5.5	5.5	5.5	5	5	4.5	5	5	6	6
3	5.5	5.5	5.5	5.5	5.5	5.5	5.5	5.5	5.5	7	7	7
4	5.5	5.5	5.5	5	5	5	5.5	5	5	5	6.5	7
5	5.5	5	5.5	6	5.5	5.5	5	5.5	5	7	6.5	7
6	4.5	5	5	4.5	4.5	4.5	5	4.5	5	6	6.5	6.5
7	4	4	4	4.5	4	4	4.5	4.5	4.5	6	6	6
8	5	4.5	4.5	4	4	4	5	5	5	5.5	6	6
9	5	4.5	4.5	4.5	5	4.5	5	5	4.5	6	6	6
10	5.5	5.5	6	5	5	5	6	5	5.5	7	7	7
11	4.5	4.5	5	6	6	5.5	5.5	5.5	5	8	7	7.5
12	5.5	5	5	5.5	5.5	5.5	6	5	5	8	7	7.5
13	5	5	5.5	5	5.5	5.5	5.5	6	5.5	8	5	5
14	6	5.5	5.5	5.5	5.5	5.5	5.5	5.5	5.5	8	6	6.5
15	5.5	5	5.5	5	5	5.5	5	5.5	5.5	8	7	6.5
16	5.5	5.5	5.5	6	5.5	5	5.5	5.5	5.5	7	7	7
17	5.5	5.5	5.5	6	5.5	5	5	5.5	5	6.5	7	6.5
18	5.5	5.5	5.5	5	5.5	5	5	5	5	7	7	7
19	5	5	4.5	5	4.5	4.5	5	5	4.5	4.5	5	5.5
20	6	5.5	5	5.5	5.5	5.5	5.5	5	5.5	4	5.5	5.5
21	6	6.5	6	6	6	6	6	5.5	6	5.5	7	6
22	6	5.5	6	5.5	5.5	5.5	6	5.5	5.5	7	6.5	6.5
23	5	5.5	5.5	6	5.5	5.5	5.5	6	5.5	6.5	6.5	6.5
24	5.5	5.5	5.5	5.5	5	5	5.5	5	5	7	6	6.5
25	4.5	4.5	4.5	4.5	4.5	4	4.5	4.5	4	7	7	6.5
26	5	4.5	4.5	4	4	4.5	4.5	4	4.5	7	7	6.5
27	4.5	4	4.5	4	4	4.5	4.5	4	4	7	7	6.5

These results demonstrate that PCA successfully extracted the core features of different chemical classes, providing a low-dimensional visualization basis for qualitative comparison of chemical differences among samples.

It should be noted that due to the limited sample size (27 samples across 9 origins, 3 per origin) and the inclusion of both GC-MS/MS semi-quantitative and LC-MS/MS relative quantitative data with different scales and variance structures, the PCA results are intended solely to visualize the relative dispersion trends of samples in chemical space. They do not constitute conclusions regarding origin discrimination or statistical inference. All descriptions of “separation” or “clustering” are direct observations from this dataset and do not represent general conclusions.

#### 3.2.2 Sample distribution patterns in chemical space

A three-dimensional score plot (Figure 2) based on the first three principal components was constructed to visualize the distribution of the 27 cigar tobacco samples in the chemical space.

**FIGURE 2:**
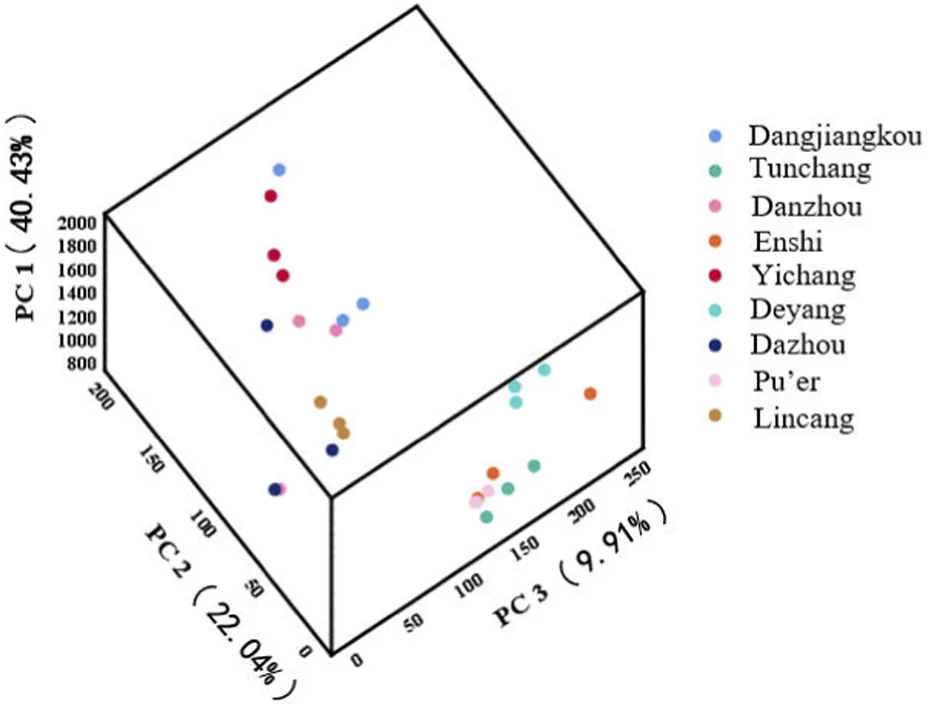
Three-dimensional PCA model.

The Sichuan samples (sample16-18 from DY; sample19-21 from DAZ) formed a relatively tight cluster on the PC1–PC2 plane, showing high within-origin consistency and clear separation from other origins along PC1. This suggests that Sichuan samples are characterized by distinct alkaloid profiles (variables with high loadings on PC1).

In contrast, within-origin dispersion was more pronounced for other regions. The three samples from DJK (sample 1–3) showed notable variability: Yunxue No. 2 (sample 1) and Dexue No. 4 (sample 3) clustered closely along PC1, while Haiyan 201 (sample 2) deviated substantially along PC2, suggesting that varietal differences may outweigh origin effects in this region. Similarly, among the PE, Yunnan samples (sample 22–24), Haiyan 201 (sample 23) and Dexue No. 4 (sample 24) nearly overlapped on PC1–PC2, whereas Yunxue No. 2 (sample 22) deviated markedly along PC3, reflecting its distinct nitrogen heterocyclic profile-consistent with its extremely low 1,2,4-triazole content (0.05 μg/mL, Table 6).

Overlapping sample positions across origins indicated chemical similarities among certain regions. Samples from DANZ (sample 7–9), YC (sample 13–15), and DJK (sample 1–3) were intermingled without clear separation by origin, suggesting considerable compositional similarity. Notably, Haiyan 201 from DANZ (sample 8) and from LC (sample 26) were positioned closely on PC2; however, due to the limited sample size (only one sample per variety per origin), this observation does not support general conclusions regardi the chemical stability of a given variety across origins.

The TC samples (sample 4–6) formed a distinct cluster in the negative PC1 and positive PC2 region, clearly separated from other origins. Consistent with the data in Table 6, these samples exhibited the lowest alkaloid levels (anabasine: 645.09–1000.54 μg/mL; nornicotine: 8.25–46.72 μg/mL) and indole content (71.24–86.98 μg/mL), indicating a uniquely low-alkaloid chemical profile.

Comparing the relative contributions of origin and variety effects, within-origin distances among different varieties (e.g., ES, sample 10–12, with indole spanning 70–98 μg/mL on PC1) sometimes exceeded between-origin distances for the same variety (e.g., Yunxue No. 2 across nine origins). This suggests that varietal effects may be comparable to or even greater than origin effects in shaping chemical composition in this dataset. However, given the limited sample size (only one sample per variety per origin), this inference requires validation with an expanded sample set.

In summary, while the PCA results reveal observable chemical differences among cigar tobacco samples from different origins and varieties, the notable within-origin dispersion among varieties and the chemical similarities across origins indicate that origins should not be simply treated as homogeneous clusters. Interpretation of origin-related chemical differences should take varietal factors into account. Given the limited sample size (3 samples per origin), these visualizations are exploratory and do not constitute conclusions regarding origin discrimination. Future studies should expand the sample size to at least 15–20 samples per origin before considering supervised classification models to assess the actual discriminability of origins.

### 3.3 Correlation analysis between chemical components and sensory evaluation

Sensory evaluation was conducted on all samples according to the criteria provided in Table 4, and the results are presented in Table 11. Spearman correlation coefficients between chemical components and sensory evaluation results were calculated using R language, and a heatmap was generated (Figure 3). The correlation strength between the top 20 chemical components (ranked by correlation magnitude) and tobacco sensory quality and combustion characteristics was systematically analyzed. The results revealed significant differences in the regulatory effects of different classes of compounds on quality attributes. In the heatmap, esters (e.g., methyl stearate (C18)), phenols (e.g., 2,4-di-tert-butylphenol), and amino acids (e.g., tryptophan, proline) exhibited strong correlations with multiple quality indicators. Based on the screening criteria described in Section 2.3, four candidate chemical markers were identified: methyl stearate, pyridinylpyrrolidone, 2,3′-bipyridine, and 2-(3-pyridinyl)-2-pyrroline. These compounds exhibited strong negative correlations with ash cohesion, delicacy, aftertaste, and aroma intensity, respectively (|r| > 0.6, P < 0.01). All four markers showed coefficients of variation (CV) greater than 30% across samples, and the observed associations were supported by biological plausibility and literature evidence. It should be emphasized that the Spearman correlation analysis between chemical components and sensory evaluation was conducted at an exploratory level; the findings are preliminary and require independent validation with larger sample sets.

**FIGURE 3:**
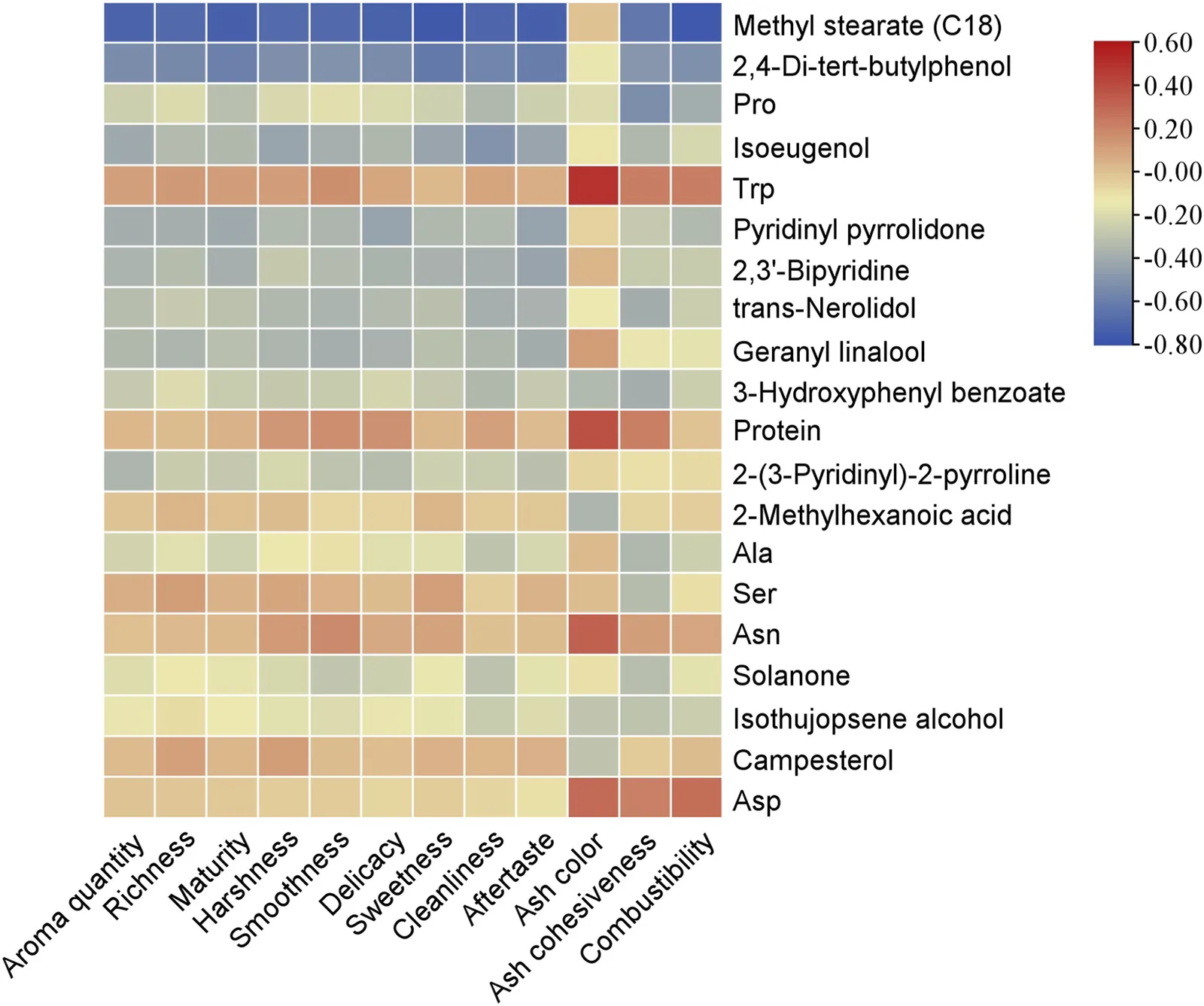
Heat map of correlation analysis between chemical components and sensory evaluation.

Ash cohesiveness of cigars represents the most intuitive external manifestation of combustion quality. The results indicated that ash cohesiveness was negatively correlated with methyl stearate (C18) (r = −0.767) (Zhu et al., 2023). Sweetness, as an important positive sensory attribute, exhibited a negative correlation with 2,4-di-tert-butylphenol (r = −0.619). As a common antioxidant, 2,4-di-tert-butylphenol may originate from tobacco processing additives; its high content significantly diminishes the natural sweetness of tobacco, necessitating strict control over its introduction pathways and residual levels during processing (Pang et al., 2023).

The study found that seven compounds, including amino acids (proline, alanine, serine), terpenoids (trans-nerolidol, geranyl linalool, isothujopsene alcohol), as well as 3-hydroxyphenyl benzoate and solanone, were negatively correlated with the “ash color” indicator (Chen C. et al., 2024; Lampman et al., 2022; Luo et al., 2025). An ideal gray ash is generally considered a hallmark of complete combustion; higher contents of these compounds tend to cause the ash to deviate from the ideal gray color, suggesting that they may interfere with ash formation by influencing the composition of pyrolysis products or combustion efficiency. Among these, although amino acids positively contribute to aroma formation (Li et al., 2024b; Chen H. et al., 2024), their excessive accumulation may lead to incomplete combustion (Deng et al., 2011). Terpenoids, as important aroma precursors, require their concentrations to be maintained within an appropriate range to balance aroma contribution with combustion appearance (Yu et al., 2025).

Combustibility was found to be co-regulated by multiple classes of compounds. Nitrogen-containing compounds such as tryptophan, protein, asparagine, and aspartic acid were positively correlated with combustibility, potentially by generating combustion-supporting nitrogenous products upon pyrolysis, thereby promoting combustion continuity (Zhu et al., 2025). In contrast, 2-methylhexanoic acid (a fatty acid) and campesterol (a sterol) were negatively correlated with combustibility; their high boiling points or pyrolysis characteristics may interfere with the combustion process (Wang et al., 2022; Zhao et al., 2013).

Cleanliness exhibited a negative correlation with isoeugenol; this compound may generate off-flavors, and high concentrations lead to reduced purity of the smoke (Bold et al., 2024). Delicacy was negatively correlated with pyridinyl pyrrolidone, suggesting that this nitrogen-containing heterocyclic compound may impart a coarse texture to the smoke, consistent with findings reported by Zhao et al. (2024). Aftertaste showed negative correlations with 2,3′-bipyridine and geranyl linalool; residual aftertaste following combustion may affect sensory pleasure (Zhu et al., 2023). Geranyl linalool, an important sesquiterpenol secondary metabolite in tobacco leaves and one of the core aroma precursors, exhibits a dual character in its role: a central positive contribution to aroma alongside potential negative effects on combustion and sensory experience (Lee et al., 2024). Aroma quantity was negatively correlated with 2-(3-pyridinyl)-2-pyrroline, indicating that this specific pyridine compound may produce suppressive odors rather than pleasant aromas at high concentrations (Zhang et al., 2024). These findings provide clear chemical targets for the precise regulation of sensory quality.

### 3.4 Limitations and future prospects

As an exploratory investigation, this study has several methodological and experimental design limitations that warrant consideration and improvement in future research.

#### 3.4.1 Sample size and experimental design

A total of 27 tobacco samples were analyzed, covering 9 origins and 3 varieties, with only 3 samples per origin. Each sample was subjected to a single injection without technical or biological replicates. Consequently, it was not possible to: (a) calculate intra-sample standard deviations to assess variability; (b) perform statistical significance tests for inter-group differences (e.g., ANOVA); or (c) draw definitive conclusions regarding origin- or variety-related differences, which should instead be interpreted as exploratory findings requiring rigorous statistical validation. To partially compensate for this limitation, systematic method validation was performed (GC-MS/MS: RSD<6.0% for six independent sample preparations and RSD <3.0% for instrumental precision; LC-MS/MS/MS: intra-day and inter-day precision RSD ≤5.0%), demonstrating the stability of the analytical workflow at the methodological level. However, method validation cannot fully substitute for replicate measurements of actual samples. Future studies should increase sample size and incorporate technical replicates (≥3 injections per sample) to enable rigorous statistical testing.

#### 3.4.2 Compound identification

GC-MS/MS identification relied solely on NIST 17 mass spectral library matching, without retention index (RI) verification or authentic standard confirmation. Therefore, all GC-MS/MS-detected compounds should be considered tentatively identified or putatively annotated rather than conclusively confirmed. This limitation is common in untargeted metabolomics studies but should be fully recognized when interpreting the results. Future work should establish a retention index system and confirm key compounds using authentic standards. In addition, the LC-MS/MS analysis employed Q3 full-scan and product ion scan modes, which offer broad compound coverage but lower sensitivity and selectivity compared to MRM mode. The detection of low-abundance nitrogen-containing compounds and the accuracy of structural assignment may be compromised accordingly. Subsequent studies should develop MRM methods for key alkaloids to achieve targeted quantitative analysis with higher sensitivity and selectivity.

#### 3.4.3 Quantification methods and matrix effects

The GC-MS/MS analysis was semi-quantitative, using a single internal standard (methyl octanoate) with an assumed response factor of 1. The reported values are therefore relative quantities suitable for inter-sample comparison rather than absolute determination. LC-MS/MS analysis employed external standard calibration without isotope-labeled internal standard correction or systematic matrix effect evaluation (e.g., post-column infusion or post-extraction addition methods). Under electrospray ionization (ESI), endogenous matrix components in tobacco extracts may cause ion suppression or enhancement, and the direct methanol extraction without a clean-up step may further exacerbate these effects. Of note, the LC-MS/MS data in this study were primarily used for semi-quantitative comparisons (PCA clustering, Spearman correlation analysis), and under identical matrix conditions, systematic matrix effects have limited influence on relative comparisons. However, interpretation of absolute concentrations of specific compounds should take these limitations into account. Future studies should introduce deuterated internal standards (e.g., nicotine-d3, nornicotine-d4) and establish systematic protocols for matrix effect evaluation.

#### 3.4.4 Method reproducibility

GC-MS/MS method validation covered instrumental precision and method repeatability but did not systematically assess inter-day reproducibility, inter-operator reproducibility, or inter-laboratory reproducibility. LC-MS/MS precision data were derived from replicate injections of standard solutions rather than from replicate analysis of actual tobacco samples. Therefore, the relative compound concentrations reported in this study represent observations obtained under single-batch experimental conditions; their stability and reproducibility across different batches and laboratories remain unclear. Future studies, with expanded sample sizes, should conduct systematic reproducibility assessments to determine whether the observed differences are stable across time and laboratories.

#### 3.4.5 Statistical analysis and modeling strategy

The PCA performed in this study was an unsupervised exploratory analysis revealing natural clustering trends of samples in chemical space, but it did not quantify the contribution of individual chemical variables to origin discrimination or sensory quality differences. Spearman correlation analysis revealed only statistical associations between variables, and the identified candidate chemical markers should not be regarded as confirmed sensory modulators. Future research, building upon the current work, will expand the sample size (15–20 samples per origin) and employ partial least squares discriminant analysis (PLS-DA) with the 16 representative chemical indicators as the predictor matrix (X) and origin categories and sensory evaluation scores as the response matrix (Y) to construct supervised classification and quality prediction models. Variable importance in projection (VIP) will be used to identify chemical components contributing most significantly to origin discrimination and sensory quality, and leave-one-out cross-validation will be employed to assess predictive performance. The causal regulatory roles of candidate markers warrant further verification through independent approaches such as sensory reconstitution experiments.

In summary, all chemical data and candidate targets presented in this study are preliminary exploratory findings that require further validation with expanded sample sizes and improved methodologies.

## 4 Conclusion

This study took a total of 27 domestic cigar tobacco leaf samples from 9 producing areas and 3 varieties as research objects. Gas chromatography-mass spectrometry (GC-MS/MS), liquid chromatography-mass spectrometry (LC-MS/MS) and biochemical kits were used to obtain multi-dimensional chemical composition data. Principal Component Analysis (PCA) was applied to reveal the distribution characteristics of samples from different producing areas and varieties in the chemical space, and Spearman correlation analysis was adopted to explore the correlations between chemical components and sensory quality indicators. The PCA score plots showed that samples from producing areas including Sichuan and Tunchang of Hainan formed relatively independent clusters, while samples from Danzhou of Hainan, Yichang of Hubei and Danjiangkou of Hubei were closely distributed, suggesting certain chemical similarities among them. Spearman correlation analysis indicated that sensory indicators such as sweetness and moistness, ash retention, combustibility, cleanliness and fineness were significantly correlated with specific chemical components including alkaloids, sugars and amino acids, which preliminarily screen out candidate chemical indicators that may be associated with the core sensory quality of cigars.

It should be noted that the sample size of this study was small, with only 3 samples collected from each producing area. The PCA results were merely exploratory data visualization and could not serve as conclusions for producing area identification. In addition, the results of correlation analysis lacked the support of independent validation sets and were hypothesis-generating. Future studies should expand the sample size of each producing area to more than 15 to 20 samples, establish independent training sets and validation sets, construct robust origin traceability and quality prediction models such as random forest and partial least squares regression, and conduct repeated verification across multiple years to evaluate the stability of the models. Meanwhile, rapid detection technologies such as near-infrared spectroscopy can be developed based on the key chemical targets identified in this study to realize real-time monitoring of tobacco leaf quality. In conclusion, this study provides an exploratory data foundation and reference of candidate chemical indicators for subsequent large-scale, confirmatory research on cigar tobacco leaf quality.

## Data Availability

The original contributions presented in the study are included in the article/Supplementary Material, further inquiries can be directed to the corresponding authors.
